# An analysis of synteny of *Arachis *with *Lotus *and *Medicago *sheds new light on the structure, stability and evolution of legume genomes

**DOI:** 10.1186/1471-2164-10-45

**Published:** 2009-01-23

**Authors:** David J Bertioli, Marcio C Moretzsohn, Lene H Madsen, Niels Sandal, Soraya CM Leal-Bertioli, Patricia M Guimarães, Birgit K Hougaard, Jakob Fredslund, Leif Schauser, Anna M Nielsen, Shusei Sato, Satoshi Tabata, Steven B Cannon, Jens Stougaard

**Affiliations:** 1 Department of Genomic Sciences and Biotechnology, Catholic University of Brasília, SGAN 916, CEP 70.790-160, Brasília, DF, Brazil; 2 Embrapa Genetic Resources and Biotechnology, C.P. 02372, CEP 70.770-900, Brasília, DF, Brazil; 3 Laboratory of Gene Expression, Department of Molecular Biology, University of Aarhus, Gustav Wieds Vej 10, DK-8000 Århus C, Denmark; 4 Bioinformatics Research Center, University of Aarhus, Høegh-Guldbergs Gade 10, Building 090, DK-8000 Århus C, Denmark; 5 Kazusa DNA Research Institute, 1532-3 Yana, Kisarazu, Chiba 292, Japan; 6 USDA-ARS and Department of Agronomy, Iowa State University, Ames, IA 50011, USA

## Abstract

**Background:**

Most agriculturally important legumes fall within two sub-clades of the Papilionoid legumes: the Phaseoloids and Galegoids, which diverged about 50 Mya. The Phaseoloids are mostly tropical and include crops such as common bean and soybean. The Galegoids are mostly temperate and include clover, fava bean and the model legumes *Lotus *and *Medicago *(both with substantially sequenced genomes). In contrast, peanut (*Arachis hypogaea*) falls in the Dalbergioid clade which is more basal in its divergence within the Papilionoids. The aim of this work was to integrate the genetic map of *Arachis *with *Lotus *and *Medicago *and improve our understanding of the *Arachis *genome and legume genomes in general. To do this we placed on the *Arachis *map, comparative anchor markers defined using a previously described bioinformatics pipeline. Also we investigated the possible role of transposons in the patterns of synteny that were observed.

**Results:**

The *Arachis *genetic map was substantially aligned with *Lotus *and *Medicago *with most synteny blocks presenting a single main affinity to each genome. This indicates that the last common whole genome duplication within the Papilionoid legumes predated the divergence of *Arachis *from the Galegoids and Phaseoloids sufficiently that the common ancestral genome was substantially diploidized. The *Arachis *and model legume genomes comparison made here, together with a previously published comparison of *Lotus *and *Medicago *allowed all possible *Arachis-Lotus-Medicago *species by species comparisons to be made and genome syntenies observed. Distinct conserved synteny blocks and non-conserved regions were present in all genome comparisons, implying that certain legume genomic regions are consistently more stable during evolution than others. We found that in *Medicago *and possibly also in *Lotus*, retrotransposons tend to be more frequent in the variable regions. Furthermore, while these variable regions generally have lower densities of single copy genes than the more conserved regions, some harbor high densities of the fast evolving disease resistance genes.

**Conclusion:**

We suggest that gene space in Papilionoids may be divided into two broadly defined components: more conserved regions which tend to have low retrotransposon densities and are relatively stable during evolution; and variable regions that tend to have high retrotransposon densities, and whose frequent restructuring may fuel the evolution of some gene families.

## Background

The legume family (Fabaceae) is one of the largest and most diverse plant families and is considered to have its origin in the tropics about 65–70 Myr ago [1]. The ability of many legumes to fix nitrogen in association with rhizobia bacteria gives them special importance in natural environments and agriculture.

The family is divided into three subfamilies, Mimosoideae, Caesalpinoideae, and Papilionoideae. Most agriculturally important species fall within two Papilionoid sub-clades that diverged some 50 Myr ago, the Phaseoloids and Galegoids. The Phaseoloids are an essentially tropical group including bean, cowpea, soya, and pigeon pea. The Galegoids are essentially temperate and include clover, pea, lentil, fava bean, chickpea and the model legumes *M. truncatula *and *L. japonicus *[1,2].

**Figure 1:**
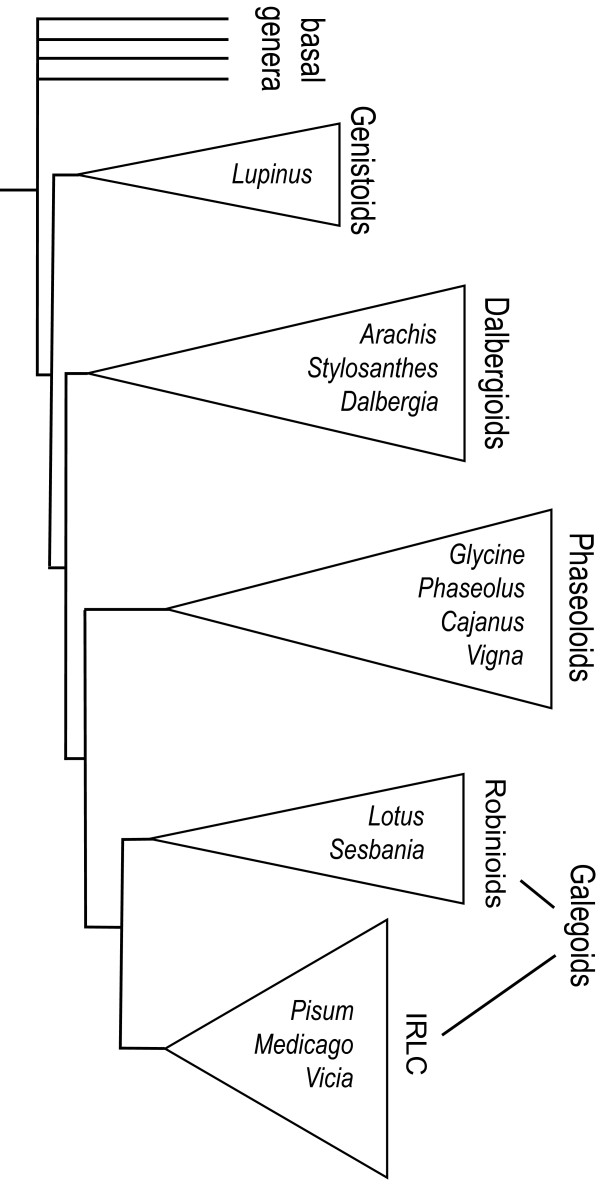
**A tree represention of the phylogeny of the Papilionoids with triangles representing the major clades, and the two subclades of the Galegoids; the Robinioids and the IRLC (plastid DNA Inverted Repeat Lacking Clade)**. Names of some notable genera are placed within the triangles. Note that *Arachis *which is a member of the Dalbergioids, represents a more basally diverged Papilionoid than the Galegoid legumes, and therefore serves as an out-group to a *Lotus vs. Medicago *comparison. The figure is a simplified and stylized phylogeny based on a tree in [2].

Two notable exceptions are lupin (*Lupinus *spp.) and peanut (*Arachis hypogaea*). They fall within the Genistoid and Dalbergioid clades respectively, which are more basal in their divergence within the Papilionoids than the Phaseoloids and Galegoids [2]. A simplified tree representation of Papilionoid phylogeny is represented in Fig. 1. Although the importance of lupin species is growing, especially in Australia, the importance of peanut is much greater. Global annual peanut production is about 36 million tonnes, with >90% being produced in the developing world. It is of great social importance, especially in Africa, and in Asia where it provides more calories than soya [3].

Cultivated peanut is an allotetraploid of recent origin, with an AB genome. Its polyploidy and low genetic diversity have hampered advances in its genetics and genomics. To avoid these problems we recently generated a map from two diploid A-type genome relatives of peanut, *A. duranensis *and *A. stenosperma*, the former being the most probable A genome donor to peanut [4], [5], [6]. In this work we aimed to align this map with the substantially sequenced genomes of *Lotus *and *Medicago *and enhance our understanding of the *Arachis *genome. In addition, because of its out-group status (Fig. 1), the results were likely to be informative about the structure and evolutionary history of legume genomes generally.

To compare genomes, orthologous loci need to be mapped in the species of interest. Using single copy genes facilitates the identification of orthologs, however, they generally have low polymorphism, making marker development difficult. The solution is to target the more variable introns. Furthermore if polymerase chain reaction (PCR) primers are designed to bind conserved sequences that flank introns, they work in a range of species and serve as a toolkit for the development of comparable "anchor markers" across a range of plant species. Previously we developed software for the design of such primers and published a set of anchor markers ideally suited to the comparison of legume genomes [7].

The level of synteny between two species depends upon the genomic restructuring events that occurred since their evolutionary divergence. Transposons have an important role in such restructuring. During evolution, transposons can undergo both massive amplifications and reductions in number, inflating and reducing the size of genomic regions. Also, the presence of transposons of almost identical sequence in multiple positions of a genome promotes recombination and genome restructuring. Transposons are divided into two classes. Class I transposons, also known as retrotransposons, have a "copy and paste" replication mechanism that involves an RNA intermediate. Class II transposons, also known as DNA transposons do not have an RNA intermediate, and usually transpose by a "cut and paste" mechanism that does not involve replication [8]. Retrotransposons are further divided into two classes, those with Long Terminal Repeats (LTR retrotransposons), and those without (non-LTR retrotransposons). LTR retrotransposons are the most abundant transposons in plant genomes. Because of this, and because of the special propensity of their terminal repeats to promote recombination events, they are considered to have a special role in plant genome evolution [9].

Here we report the placement of 102 anchor markers on the map of the *Arachis *A-genome and analyses of synteny of *Arachis *with *Medicago *and *Lotus*. In addition, we investigated if there was a correlation between genome conservation and transposons. Finally, we briefly investigated gene distribution in relation to synteny and retrotransposon distribution.

## Methods

### Plant material

Genetic mapping was done with a previously described F_2 _population of 93 plants derived from a cross of *A. duranensis *(K7988) with *A. stenosperma *(V10309) [4].

### Marker development

Legume anchor markers were developed essentially as described in [7]. Key features of the marker development were:

i) Identification of ESTs from multiple legume species, usually *Lotus*, soya and *Medicago*, with single strong blast detected sequence similarites against all predicted *Arabidopsis *proteins and the alignment of these ESTs.

ii) Alignment of ESTs to a corresponding genomic region from *Lotus *or *Medicago *and inference of intron positions.

iii) Identification of conserved intron-flanking sequences, and design of primers to bind these conserved sequences.

The rationale behind this procedure is that:

i) Markers to unique sequences within a genome facilitate the comparison of genetic maps, and genes that are single copy in *Arabidopsis *have a high probability of being single copy in legume genomes (also see results).

ii) Introns are more variable than coding regions, and therefore they are better for marker development.

iii) Primers that bind to sequences that are conserved are more likely to be transferable to other species.

The primers were used in PCR with the progenitors of the *Arachis *mapping population. Polymorphisms were identified by size- or sequence variation. In the latter case, most markers developed were Cleaved Amplified Polymorphic Sequence (CAPS) or derived Cleaved Amplified Polymorphic Sequence dCAPS [10].

Other markers used in map construction have been [4], or will be, published elsewhere (unpublished data).

### Genetic Mapping

Linkage analysis was done with Mapmaker Macintosh version 2.0 [11] essentially as described in [4].

### Analysis of synteny

Points of correspondence of *Arachis *with the model legumes were assigned by using *Arachis *marker sequences as queries in blast searches against the genome sequence and genetic mapping for *Lotus *and, for *Medicago*, by blast similarity searches against the genome sequence using CViT blast (Chromosome Visualization Tool, ). For legume anchor markers, and other markers with a single strong blast detected sequence similarity to *Arabidopsis *predicted proteins in addition to the the *Arachis *nucleotide sequence, we also used the *Arabidopsis *protein sequence in blastn, tblastn and blastp searches. A single strong blast detected sequence similarity was defined as E-value for the best blast alignment being at least 10^6 ^times lower than the E-value for the second best blast alignment.

Most of the *Lotus *Bacterial Artificial Chromosome/Transformation competent Bacterial Artificial Chromosome (BAC or TAC) clones identified in blast searches using *Arachis *marker sequences had been positioned on the *Lotus *map based on the intraspecific cross between ecotypes Gifu and Miyakojima MG-20 [12]. Where a map position was absent, or only a whole genome shotgun or EST sequence was available, the corresponding BAC/TAC clone was isolated and/or sequenced and mapped in Gifu × MG-20 and/or in *L. filicaulis *× *L. japonicus *Gifu [13]. All map positions are given with respect to the former map. Tandem Repeat Occurrence Locator  was used for identifying the microsatellites for genetic mapping.

Tables of correspondences between *Arachis *markers and the model legume genomes were used in a Microsoft Excel file, together with logical functions, to create plots of the *Arachis *genetic map against *Lotus *and *Medicago *(Additional file 1).

**Figure 2:**
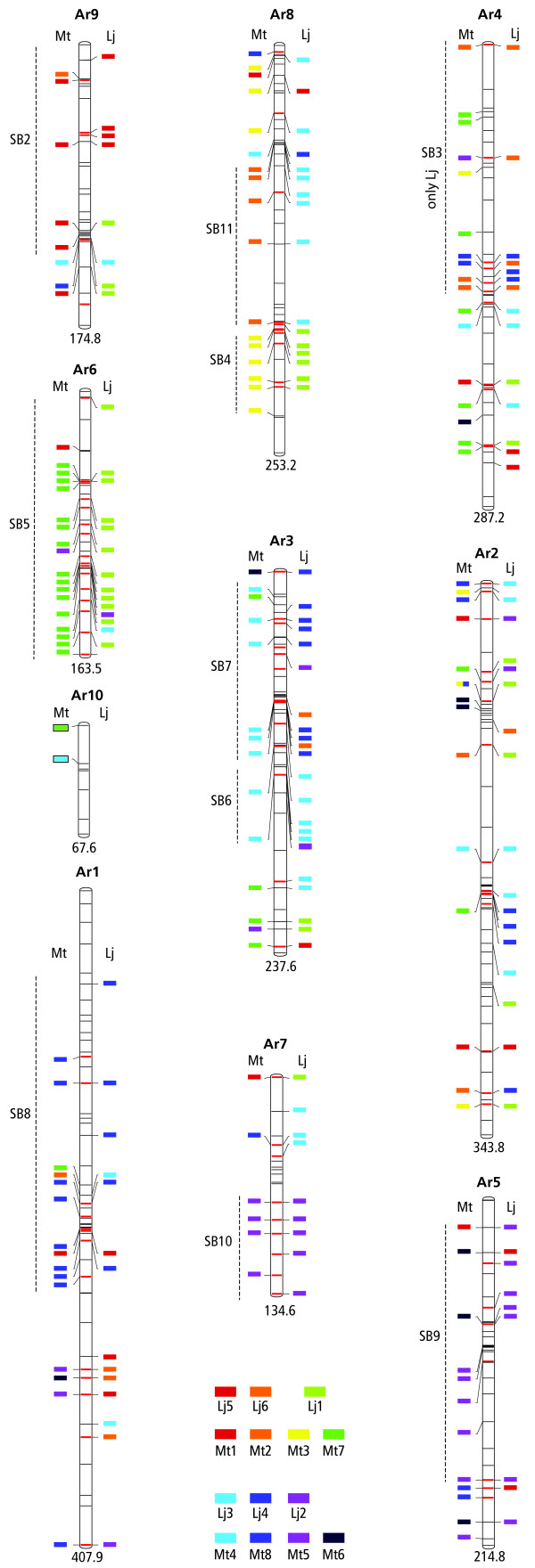
***Arachis *LGs with affinities to *Lotus *and *Medicago *chromosomes represented as colored blocks, and synteny blocks (SBs) indicated**. *Arachis *LGs are numbered according to [4] with size in cM indicated below. Marker positions are indicated as horizontal lines across LGs, anchor markers as red lines, other markers as black lines. Colors were assigned to the model species chromosomes so that syntenous chromosomes are represented by corresponding colors. SBs are numbered according to [14], with the addition of SB11 identified in this study. A full genetic map is in Additional file 2.

In addition, to visualize a comparison of the *Arachis *genome simultaneously with *Medicago *and *Lotus*, colored coded blocks representing *Arachis *marker correspondences with *Lotus *and *Medicago *were placed along the sides of *Arachis *LGs. Colors were assigned to the model legume chromosomes following a rainbow spectrum along the X and Y axes of the previously published *Lotus vs. Medicago *genome plot [14], in such a way that corresponding groups of *Lotus *and *Medicago *are represented by corresponding or neighboring colors in the spectrum (Fig. 2 and Additional file 2).

**Figure 3:**
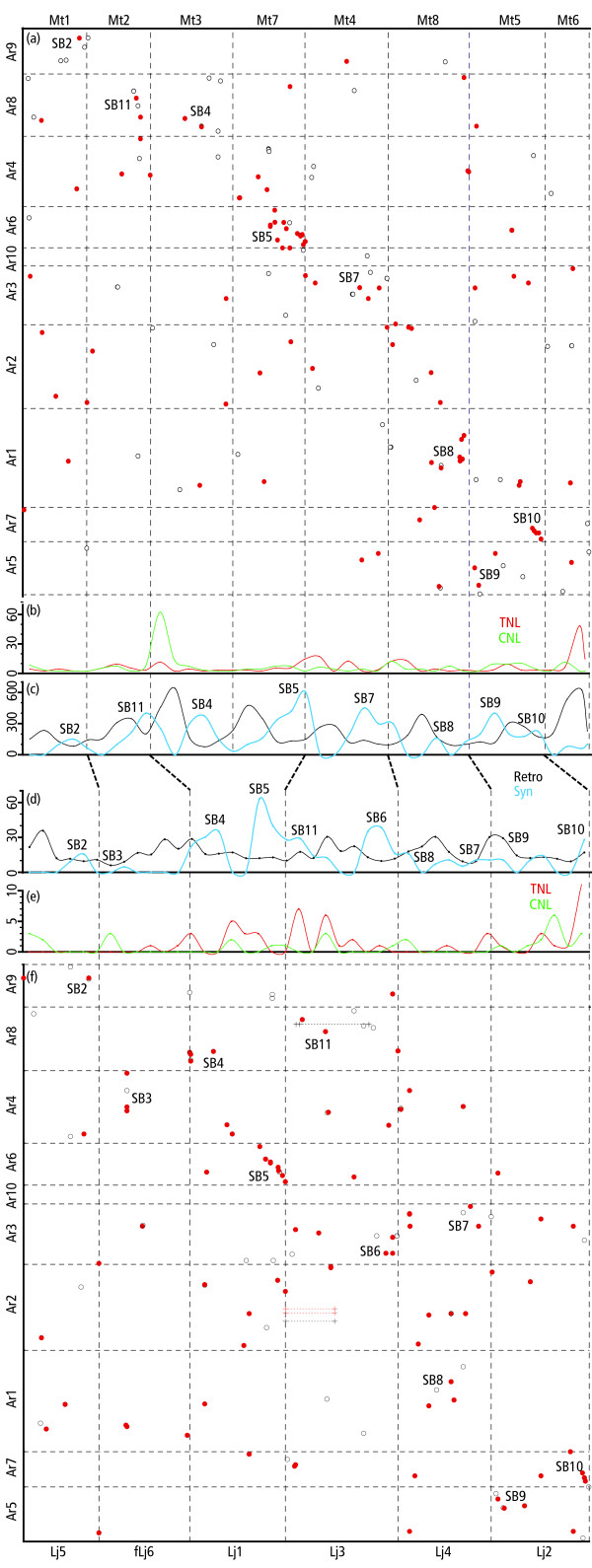
**Genome plots of *Arachis vs*. model legumes, and graphs of, synteny with *Arachis*, retrotransposon, and resistance gene homolog distributions for the model legumes**. In the plots, marker correspondences are solid red dots for top Blastp and tBlastn homologies of anchor markers, and hollow black dots for top Blastn homologies of all sequence characterized markers. Chromosome orders and numbering of SBs (with addition of SB11) the same as in [14], allowing direct comparisons. Corresponding *Medicago *chromosomes and *Lotus *LGs are joined with slanted lines in the middle of the figure. For interactive versions of the plots see Additional file 1. (a) Genome Plot of *Arachis *(cM) *vs. Medicago *(bp). (b) Density of tBlastn detected sequence similarities (E-values < 1E^-20^) for the TNL (red line) and CNL (green line) subclasses of resistance gene homologs plotted along the *Medicago *genome. Values averaged over 6 Mbp window size. High densities of resistance gene homologs and retrotransposons coincide. (c) Black line: density of Blastn detected sequence similarities (E-values < E^-60^) for retrotransposons plotted along the *Medicago *genome. Cyan-blue line: synteny score of *Medicago *with *Arachis *scaled by multiplying by 100. Values averaged over 6 Mbp window size. SBs occur in regions of low retrotransposon density. (d) Percentage genome coverage of retrotransposons plotted along the *Lotus *genome (values averaged over 10 cM window size). Cyan-blue line: synteny score of *Lotus *with *Arachis *multiplied by 7. (Values averaged over 10 cM window size). SBs tend to occur in regions of low retrotransposon coverage. (e) Density of resistance gene homolog encoding sequences, TNL (red) and CNL (green), plotted along the *Lotus *genome (averaged over 10 cM window size). Clusters of resistance gene homologs and retrotransposons coincide. (f) Genome Plot of *Arachis *(cM) *vs*. *Lotus *MG-20 (cM). Markers mapped to intervals are plotted as horizontal lines. fLj indicates that the *Lotus *chromosome has been inverted. The reference orientation of Lj5 has recently been inverted, thus Lj5 in this plot is equivalent to fLj5 in [14].

A synteny score was assigned to each point of correspondence between *Arachis *and the model legumes according to its number of neighbors within a continuous color block. For instance, the top marker in *Arachis *linkage group 9 (Ar9) has a *Arachis-Lotus *synteny score of 3, and the last marker on Ar6 has a *Arachis-Medicago *synteny score of 8 (Fig. 2 and Additional file 2). Synteny scores of markers were plotted along the model genomes, with values averaged over 6 Mbp windows for the *Medicago *genome sequence, and over 10 cM windows for the *Lotus *genetic map (Fig. 3c, 3d).

### Analysis of transposon distributions

*Lotus *transposon sequences from a Kazusa *Lotus *transposon data set were used as a target blast database in RepeatMasker , run on the *Lotus *BAC/TAC sequences. Results were summarized as percentage sequence coverage for Class I and Class II transposons for each BAC/TAC using Perl . This result, together with the genetic map positions of the BAC/TACs was used to plot the retrotransposon distributions within the *Lotus *genome with values averaged over 10 cM window size (Fig. 3d).

*Medicago *Class I and Class II transposon sequences from GIRI  were submitted to CViT blast. The output was used to plot transposon distribution along the *Medicago *genome with values averaged across a 6 Mbp window size.

### Plant disease resistance gene homolog distributions

*Lotus *Kasuza gene annotations for Coiled-Coil – Nucleotide Binding Site – Leucine Rich Repeat (CNL) and *Drosophila *Toll and mammalian Interleukin (IL)-1 receptor – Nucleotide Binding Site Leucine Rich Repeat TNL gene classes within BAC/TAC sequences were used together with the genetic map positions of the BAC/TACs to plot the gene class distributions with values averaged across a 10 cM window size (Fig. 3e).

For *Medicago*, consensus sequences of the extended NBS domains from the CNL and the TNL subfamilies of plant disease resistance genes from [15] were used in CViT blast searches, and the distribution of blast detected similarites (E-value ≤ 1E-20) plotted on the *Medicago *genome with values averaged over 6 Mbp window size (Fig. 3b).

## Results

### Marker development

Introns in anchor genes showed high levels of polymorphism between the progenitors of the *Arachis *mapping population (*c. *1 single nucleotide polymorphism/90 bp and 1 insertion/deletion polymorphism/1.6 kbp). Eighty seven intron-based anchor markers were developed and genotyped, most being CAPS or dCAPS (Additional file 3).

### Mapping

Using a minimum LOD score of 3.0 and a maximum recombination fraction (θ) of 0.35, 351 markers mapped into 10 linkage groups (LGs). Of the 87 legume anchor markers genotyped, 81 were assigned to a LG. In addition, 21 other sequence characterized mapped markers were homologs of single copy genes in *Arabidopsis *and thus were effective anchors, making a total of 102 mapped anchor markers. Anchor markers were distributed through all of the LGs, except the smallest, Ar10. Most other mapped markers are microsatellites or sequence characterized AFLP.

LGs were numbered according to [4], however, the inclusion of new markers resulted in 10 LGs, corresponding to the number of chromosome pairs in *Arachis*, instead of the previous 11.

### Synteny analysis

Of the legume anchor markers, only six (Leg050, Leg168, Leg33MGm, Leg20MGm, Leg069, Leg034), had strong multiple affinities to the *Lotus *genome and four to the *Medicago *genome (LegTC987, Leg202, Leg304, Leg299). This clearly implies that anchor marker genes (represented in a single copy in *Arabidopsis*) tend to also be represented as single copy genes in the model legumes making them ideal markers for the comparison of genomes.

Using sequence information from the 102 anchor markers and 228 other mapped and sequence characterized markers, we were able to make 128 and 126 points of correspondence between *Arachis *and *Medicago *and *Lotus *respectively.

Following the previously published dot plot presentation of genome similarity between *Lotus *and *Medicago *[14], *Arachis *homologies with the model legumes mostly corresponded to regions within the main axis of the *Lotus vs. Medicago *genome plot. The color scheme used in Fig. 2 (and Additional file 2), where corresponding model legume chromosomes match, facilitates the simultaneous visualization of *Arachis *homologies with the model legumes. For instance, *Medicago *chromosome 1 (Mt1) and *Lotus *LG5 (Lj5) are syntenic, are the first in their genetic map/genome orders in the dot plots, and thus have a red color code. Ar9 has a predominance of correspondences with Mt1 and Lj5 which is therefore also placed first in the order for the dot plots. The predominance of "red" associations of Ar9 in Fig. 2 (and Additional file 2) makes this easily visible. Similarly, progressing through Ar8, Ar4, Ar6, Ar10, Ar3, Ar2, Ar1, Ar7 and Ar5 the main affinities with the model legumes roughly follow through the spectrum with the last, Ar5, having predominantly violet (Lj2, Mt5) affinities. The main exception to this is the lower region of Ar8 which has Mt2 – Lj3 affinities. This region corresponds to the main off-axis region of synteny in the *Arachis vs. Lotus *plot, and which we named synteny block 11 (SB11).

The affinities of *Arachis *LGs and *Lotus *chromosomes in the plot shows a diagonal tendency (Fig. 3f), although a number of factors make the diagonal less clear than for the *Arachis vs. Medicago *plot (see below): i) Areas of suppressed recombination in Lj6, and the top of Lj1, creates vertical lines of points in the Ar4 – Lj6 and Lj1 – Ar8 squares, if physical instead of genetic distances could have been used, diagonal lines or clusters could well have been formed. ii) There is a substantial off-axis Ar8 – Lj3 cluster in the plot (SB11), this cluster presumably reflects an ancient translocation event, and apparently corresponds to an off-axis area (Mt2 – Lj3) in the *Lotus vs. Medicago *plot [14]. iii) Lack of resolution in some regions of the genetic maps means that some points are superimposed, for instance the *Arachis vs. Lotus *region SB2 appears to be supported only by two points in the plot, but in fact is supported by four (Fig. 2 and Additional files 1 and 2). Nevertheless, for most of the *Arachis *LGs the overall affinities are clear, co-linearities are visible between Ar6 and the lower portion of Lj1, and between Lj2 and Ar7. Some areas of the plot show double associations: for instance Ar6 and Ar2 with the bottom of Lj1; and the bottom of Ar2 and the top of Ar1 with the middle of Lj4.

Points of correspondence between *Arachis *and *Medicago *are all from blast searches of the pseudomolecules of *Medicago *genome. This approach is an alternative to the direct comparison of linkage positions used in the *Arachis-Lotus *comparison. These two somewhat different approaches were used because of the different forms of the data from the genome projects, and because the *Lotus vs. Arachis *comparison was partly based on blast searches against genome data and partly on genetic mapping. Correspondences between *Arachis *LGs and *Medicago *chromosomes were found in seven of the 10 *Arachis *LGs. Of the remaining three, two (Ar4 and Ar2) have "shattered", or fragmented, synteny. The remaining Ar10 shows no clear affinities, although it has been tentatively assigned a position within the genome plot according to two blast detected sequence similarities with *Medicago*. The plot of *Arachis *LGs *vs. Medicago *chromosomes shows a diagonal tendency (Fig. 3a). There are two apparent regions of co-linearity: lower region Mt2 – central region Ar8; lower region Mt5 – lower region Ar7. In the remainder of the plot, points often cluster within squares. The clearest example being Ar6 – Mt7: intra-chromosomal rearrangements since the *Arachis *– *Medicago *evolutionary divergence have disrupted co-linearity, but a clear Ar6 – Mt7 correspondence is preserved.

The plots of *Lotus vs. Medicago *[14], of *Arachis vs. Lotus*, and of *Arachis vs. Medicago *(Fig. 3a, 3f) show interesting similarities. For instance, Ar6, the bottom of Lj1 and the bottom of Mt7 all show good synteny with each other – the region has maintained high levels of synteny over some 55 Myr of evolutionary divergence. In contrast, the bottom of Ar4, the top of Mt7, and the middle of Lj1 all show relatively poor synteny with each other. This pattern is repeated and most of the synteny blocks (SBs) visible in the *Lotus vs. Medicago *plot are visible in *Arachis vs. Medicago *and *Arachis vs. Lotus *plots (Figs. 2, 3a, 3f). Furthermore, most model legume chromosome regions that lack detectable synteny in the model legume plot, also lack synteny in the *Arachis vs. *model legume plots. Overall, in the *Arachis vs. Lotus *plot the following SBs are detected, 2, 3, 4, 5, 6, 7, 8, 9, 10. For the *Arachis vs*. *Medicago *plot the following SBs could be identified, 2, 4, 5, 7, 8, 9 and 10. SB1 which consists of synteny between the upper regions of Mt1 and Lj5 could not be detected in either of the *Arachis *plots. (Figs. 2, 3).

In addition to the synteny blocks previously identified in the *Lotus vs. Medicago *comparison, an unnumbered off-axis region of synteny visible in the *Lotus vs*. *Medicago *plot was also apparent in the *Arachis vs. *model legumes plots. This region consisting of Ar8 – Mt2 – Lj3 affinities was named SB11.

An Excel file with all marker data, synteny information and genome plots of *Arachis vs*. *Medicago*/*Lotus *is available as Additional file 1. We hope that the file allows the reader to get a good 'feel' for the data, because within the file, parameters can be changed and the effects on the plots visualized.

### Analysis of transposon, single copy genes and disease resistance gene homolog distributions and synteny with *Arachis *in *Lotus*

DNA transposons on average cover 10.4% of both mapped and unmapped *Lotus *BAC/TACs. The distribution is uneven, varying from 6.1% to 14.2% when averaged over 10 cM windows. Retrotransposons are more abundant, covering on average 19.4% of mapped TACs/BACs and 25.6% of unmapped clones, and are more unevenly distributed, varying from 5.9% to 35.8%, averaged over 10 cM windows. There is a negative correlation between retrotransposon coverage in the genome and synteny score with *Arachis *(values averaged over 10 cM window size; r = -0.22; Fig. 3d). Retrotransposon coverage is very high (*c. *30%) in *Lotus *in exactly the region of the missing synteny block SB1 (Fig. 3d).

To study gene distributions we focused on two groups of genes with distinct evolutionary pressures: single copy genes, the distribution of which was already apparent from the distribution of anchor marker correspondences, and one of the highest copy number and best characterised of plant gene families, nucleotide binding site encoding resistance gene homologs; Coiled-Coil – Nucleotide Binding Site – Leucine Rich Repeat (CNL) and *Drosophila *Toll and mammalian Interleukin (IL)-1 receptor – Nucleotide Binding Site Leucine Rich Repeat (TNL) gene sub-classes [15].

In *Lotus *the density of anchor marker, and hence of single copy gene, correspondences with *Arachis *is not evenly distributed; synteny blocks tend to have higher densities of anchor markers and hence single copy genes (anchor markers, are shown as red lines across LGs in Fig. 2, as red marker names in the expanded version of Fig. 2 presented in Additional file 2, and by solid red dots in Fig. 3a, 3f). The analysis of resistance gene homologs showed that, in the currently available *Lotus *genome sequence [16] there are 54 CNL and 174 TNL genes encoding disease resistance gene homologs. Of these, 33 CNL and 54 TNL are in mapped TACs and BACs, and the remainder in unmapped clones or whole genome sequence. These genes are not evenly distributed and tend to form clusters, for instance in the upper region of Lj3 and the lower region of Lj2. A number of these clusters are located within regions of high retrotransposon densities. For instance, in the upper region Lj3 there are 13 TNL and 4 CNL encoding genes and in the top of Lj3 there are five CNL encoding genes (Fig. 3e). Overall there was no correlation of CNL genes and retrotransposons, however, the correlation coefficient of TNL genes and retrotransposons was 0.2.

### Analysis of transposon and disease resistance gene homolog gene family distributions and synteny with *Arachis *in *Medicago*

In *Medicago *there are about 2.8 retrotransposons to each DNA transposon. LTR retrotransposons outnumber non-LTR retrotransposons by about 16 fold. Retrotransposons are very unevenly distributed in the *Medicago *genome sequence. There is a clear tendency that *Medicago *regions with high synteny scores with *Arachis *have low retrotransposon density, and that regions with low synteny scores have high retrotransposon densities (Fig. 3c). The Correlation coefficient of synteny score and density of blast detected retrotransposon sequence similarities (E-value ≤ 1E-60) per 6 Mb of *Medicago *genome was -0.35. Retrotransposon densities are moderately high in *Medicago *in exactly the region of the missing synteny block SB1 – the top of Mt1 (Fig. 3a, 3c, ref [14]).

In *Medicago*, as was the case in *Lotus*, the density of the anchor marker correspondences with *Arachis *is not evenly distributed; there is a trend that synteny blocks have higher densities of anchor markers, and hence of single copy genes (Fig. 2, 3 and Additional files 1 and 2).

In the *Medicago *genome sequence, there are 177 CNL and 156 TNL encoding sequences [15]. Of these, four CNLs and two TNLs are on unmapped BACs. The genes are not evenly distributed and tend to be clustered. Particularly striking are two superclusters, much higher in copy number than any cluster identified in *Lotus *(Fig. 3b, note that the scale for resistance gene homolog distributions in *Medicago *and *Lotus *are different). One of these superclusters is mostly of CNL encoding sequences at the top of Mt3, and one of TNL encoding sequences at the bottom of Mt6 [15]. The Mt3 supercluster contains 73 CNL and 9 TNL encoding sequences, the Mt6 supercluster contains 57 TNL encoding sequences. Other lower copy number clusters are also apparent, for instance on Mt4, Mt8 and Mt5, these clusters are more similar in copy number to the clusters observed in *Lotus*. The correlation coefficients of TNL and CNL encoding sequences with retrotransposons are 0.39 and 0.33 respectively.

### Plots of the overall profiles of synteny with *Arachis *and retrotransposons in the *Medicago *and *Lotus *genomes

The comparison of the plots of synteny score with *Arachis *and retrotransposon density along the genomes of *Medicago *and *Lotus *shows similarites in the patterns of the peaks within corresponding groups (Fig. 3c, 3d). The profiles of synteny with *Arachis *plotted along the model legume genomes is most conserved, with a similarity of peaks (corresponding to synteny blocks) both with respect to positions and even relative heights. The main exception being that the order of the synteny blocks that are off-axis in the genome plots (SB7 and SB11) is changed. These rearrangements presumably correspond to chromosome translocation events in one of the three evolutionary lines leading to the model legumes and *Arachis*. Peak structure of retrotransposon densities is similar especially when considering the time of evolutionary divergence between *Lotus *and *Medicago*. However, relative peak heights of retrotransposon densities are markedly different, for instance while the highest retrotransposon concentrations in *Medicago *are at the top of Mt3 and the lower portion of Mt6, the highest densities in *Lotus *are in the non-corresponding groups Lj5, Lj3 and Lj4.

## Discussion

Integrated maps that link crop and model plants allow knowledge gained from independent research on different plants to be accumulated [17]. This work links the genetic map of peanut, one of the world's most important grain legumes, to the model legumes *Lotus *and *Medicago*, a first step for its inclusion in an integrated genetic system for legumes.

Genome restructuring progressively breaks down syntenic relationships between species over evolutionary time. In addition, whole genome duplications occur periodically during plant evolution, followed by progressive diploidization [18]. These events split and obfuscate syntenic relationships. For the legumes, useful levels of synteny have been shown between the Galegoid models and the Phaseoloids, which diverged some 50 Myr ago [19], [20], [21]. However, between the models and the more basally diverged Genistoid lupin, syntenic relationships are more complex [22,23]. The divergence of the Dalbergioid clade to which *Arachis *belongs is placed at a similar, or slightly later, date in evolutionary time than the divergence of the Genistoids. Therefore the degree to which we could detect macro-synteny between the models and *Arachis *was uncertain.

Initial inspection of the *Arachis vs. *model legume plots show surprising degrees of synteny considering the time of species divergence. Although there are some regions of double affinities between *Arachis *and the model legumes, most synteny blocks have a single main affinity and not two affinities interleaved (Figs. 2, and 3). These patterns indicate that the last universal legume whole genome duplication predated the divergence of *Arachis *from the Galegoids and Phaseoloids sufficiently that the common ancestral genome was substantially diploidized. Synteny at the macro-level between *Arachis *and the model legumes will be useful for many genomic regions.

The different cross-species plots showed fascinating similarities, and the power of the *Arachis *out-group (Fig. 1) allows new inferences to be made: In the *Lotus vs. Medicago *genomic plot [14] distinct conserved synteny blocks and non-conserved regions are observed. To explain this, we could hypothesize rearrangements/deletions within the non-conserved regions, either in *Medicago *or *Lotus*, or in both. Although either explanation is possible, the philosophical principle of Ockham's razor guides us to prefer the simplest explanation: that regions lack synteny due to disruption in *Medicago *or *Lotus *but not both. However, with the addition of the *Arachis *out-group, the power of inference is increased: we see similar patterns of synteny (and disruption) in all possible species by species comparisons (with the notable exception of SB1). Therefore, the evidence from the *Arachis *out-group strongly argues against the simplest explanation for patterns of *Medicago *and *Lotus *synteny and disruption. The inference, instead, is that certain legume genomic regions are consistently more stable during evolution than others. Additional evidence for this was found in our recently completed study, where 104 anchor markers mapped in bean are used to detect genomic regions that are syntenic with *Lotus, Medicago *and *Arachis*. In this study, large syntenic segments are also found to be conserved between all species. These syntenic segments correspond to synteny blocks 2, 3, 4, 6, 7, 8, 9, 10 and 11 (the latter consisting of a block of bean LG5 – Lj3 – Mt2 – Ar8 associations). SB1 and SB5 are also evident, but the former is small, covering only 2.3 cM in LG1 of bean, and the latter is fragmented into three sections covering bean LG1 and LG6 ([21]; *Arachis vs. *bean marker correspondences from this reference are also summarized in Additional file 1).

We sought an explanation for these observations and began by analyzing transposon distributions in *Lotus *and *Medicago*. We found that retrotransposons are very unevenly distributed in both the model legume genomes and that the retrotransposon-rich regions tend to correspond to variable regions, intercalating with the synteny blocks (Fig. 3c, 3d). This tendency is particularly evident for *Medicago*, its higher retrotransposon content [14], and higher proportion of anchored BACs compared to *Lotus *may account for this. Overall, considering the time of evolutionary divergence, the patterns of synteny blocks, variable regions and retrotransposon distributions are substantially similar. In addition, it is notable that SB1, which is conserved between *Medicago *and *Lotus*, but was not evident between *Arachis *and the model legumes, is positioned on local peaks in the densities of retrotransposons in the model legumes. Considering this, we suggest that the euchromatic gene space of the model legumes, and by inference possibly most Papilionoid legumes, can be divided into two broadly defined components: regions that remain relatively stable, and regions that experience high rates of genome restructuring. The former tend to be syntenic across taxa and to have low retrotransposon densities, and the latter tend to show little synteny, and to have high retrotransposon densities. The proposed genome model is similar to the pan-genome concept, originally from bacteria but recently suggested for plants [24]. It should be noted that this model appears consistent with the data used here, from diploid genomes. However, plants which have undergone rapid genome restructuring after polyploidy, such as soya, may differ.

In comparing syntenic and more variable regions, another trend seems clear. Variable regions have lower densities of anchor-marker correspondences and therefore single copy genes (Figs. 2, 3a, 3f). However, regions without synteny are not simply "holes" in the dot plot, because there are correspondences in these regions; but they are scattered. In contrast to the low concentrations of single copy genes, some of the retrotransposon rich regions in the model legumes host high densities of some genes in multigene families, and we illustrated this with the plant disease resistance gene homologs.

The fast evolving nature of the repetitive fraction of the genome has been documented in many plant species. This evolution involves rapid expansions and reductions in transposon numbers [25], such that, for instance, even closely related species of *Arachis *can be distinguished using whole genome *in-situ *hybridization [6]. Therefore, it seems likely that the restructuring within the variable regions, which tend to be transposon rich, frequently includes amplification of some sequences and elimination of others ("birth and death"). Considering this, it would be expected that natural selection would tend to select against single copy or essential genes, and high densities of transposons inhabiting the same genomic regions. On the other hand, it may be expected that the co-localization of certain fast-evolving multigene families and high densities of transposons could be advantageous. The presence of high densities of resistance gene homologs, a gene family for which rapid birth and death, and frequent diversifying selection has been well established, in some of the variable retrotransposon rich regions, supports this view. A detailed phylogenetic analysis of NBS-LRR genes in *Medicago *also provides support to the hypothesis that restructuring in the variable regions has driven the evolution of some resistance gene clusters; NBS-LRR encoding genes in retrotransposon rich regions are, on average, more recent in origin, and have more unusual domain rearrangements than those in synteny blocks [15].

Retrotransposon rich genomic regions may play a similar role in legumes as in trypanosomes, where they interrupt synteny and are associated with gene family expansions and the evolution of new gene diversity [26,27]. The legume retrotransposon rich regions may also be similar to pericentromeres, exceptional genomic regions that are also retrotransposon rich: in animals they contain segmental duplications implicated in gene creation, and in plants they harbor rearrangements and insertions uncommon in euchromatin [28]. However, the size of the retrotransposon rich regions described here, extending in some cases to entire or nearly entire euchromatic chromosome arms, and the association of some of them with disease resistance genes, seems notable. For applied science, the presence of clusters of resistance gene homologs in regions of low synteny also has important implications. Synteny between genomes may often not enable predictions of the locations of orthologous resistance genes, although the genome model presented here may aid in the identification of the resistance genes for which synteny is more likely to be preserved.

## Conclusion

In summary, we have presented evidence that the last whole genome duplication within the legumes preceded the divergence of *Arachis *and the model legumes. We also show that levels of macrosynteny between *Arachis *and the model legumes within ten synteny blocks will be useful for studies of *Arachis*, for instance, to aid in gene cloning and candidate gene identification. We also show that the retrotransposon profile in the model legumes is negatively correlated with the maintenance of macrosynteny during legume evolution, and that a very substantial percentage of gene space lies outside identifiable synteny blocks. We suggest that the gene space in Papilionoids may be divided into two broadly defined components: regions that are more conserved in evolution which tend to have low retrotransposon densities; and variable regions that tend to be retrotransposon rich, and whose frequent restructuring may fuel the evolution of plant disease resistance genes and perhaps other multigene familes.

## Authors' contributions

DJB participated in conceiving and coordinating the study, analysis of data and was the main author responsible for writing the manuscript. MCM carried out the genotyping and map construction in *Arachis*. LHM participated in, and coordinated anchor marker development. NS participated in anchor marker development in *Lotus *and *Arachis *and did genetic mapping in *Lotus*. SCMLB participated in anchor marker development and genotyping and the development and maintenance of mapping populations. PMG participated in the development and maintenance of mapping populations and genotyping in *Arachis*. BKH participated in anchor marker development in *Arachis*. JF and LS designed universal legume anchor marker primers. AMN participated in anchor marker development in *Arachis*. SS and ST carried out the sequencing of *Lotus *BACs and the analysis of resistance genes and retrotransposons in *Lotus*. SBC helped interpret the data. JS conceived and co-coordinated the study and participated in interpretation of the data. MCM, LHM, NS, SCMLB, PMG, BKH, SBC and JS also helped to elaborate the manuscript. All authors read and approved the manuscript.

## Supplementary Material

Additional file 1 **Analysis of synteny of *Arachis *with *Lotus *and *Medicago*.** This file contains the *Arachis *genetic map, marker sequences, outputs from blast similarity searches against *Lotus *and *Medicago*, and the analyses of synteny. The results are presented in two forms, a color coded sheet of marker correspondences between *Arachis*, and *Lotus *and *Medicago *(also the alignments against bean from Hougaard et al. 2008 are shown), and dot plots of *Arachis vs. Lotus *and *Arachis vs. Medicago*. The dot plots are interactive and the reader can change the plot parameters and visualize the results. Click here for file

Additional file 2 **Full version of genetic map represented in Fig. **2. This file contains a full version of the genetic map shown in Fig. 2. *Arachis *linkage groups are shown with marker names and with affinities to *Lotus *and *Medicago *chromosomes represented as colored blocks, and with synteny blocks indicated. Click here for file

Additional file 3 **Legume comparative anchor marker supplementary information.** This file contains marker information for all Universal legume anchor markers used in the study. The information includes the primer pairs designed to amplify orthologous regions from a wide range of legume species. Click here for file
