# Supplementary material for: An analysis of synteny of Arachis with Lotus and Medicago sheds new light on the structure, stability and evolution of legume genomes
Source: BMC Genomics. 2009 Jan 23;10:45. doi: 10.1186/1471-2164-10-45 (PMC2656529; doi:10.1186/1471-2164-10-45)
Supplement: Additional file 2 — Full version of genetic map represented in Fig. 2. This file contains a full version of the genetic map shown in Fig. 2. Arachis linkage groups are shown with marker names and with affinities to Lotus and Medicago chromosomes represented as colored blocks, and with synteny blocks indicated. [file 1471-2164-10-45-S2.ppt]

## Slide 1
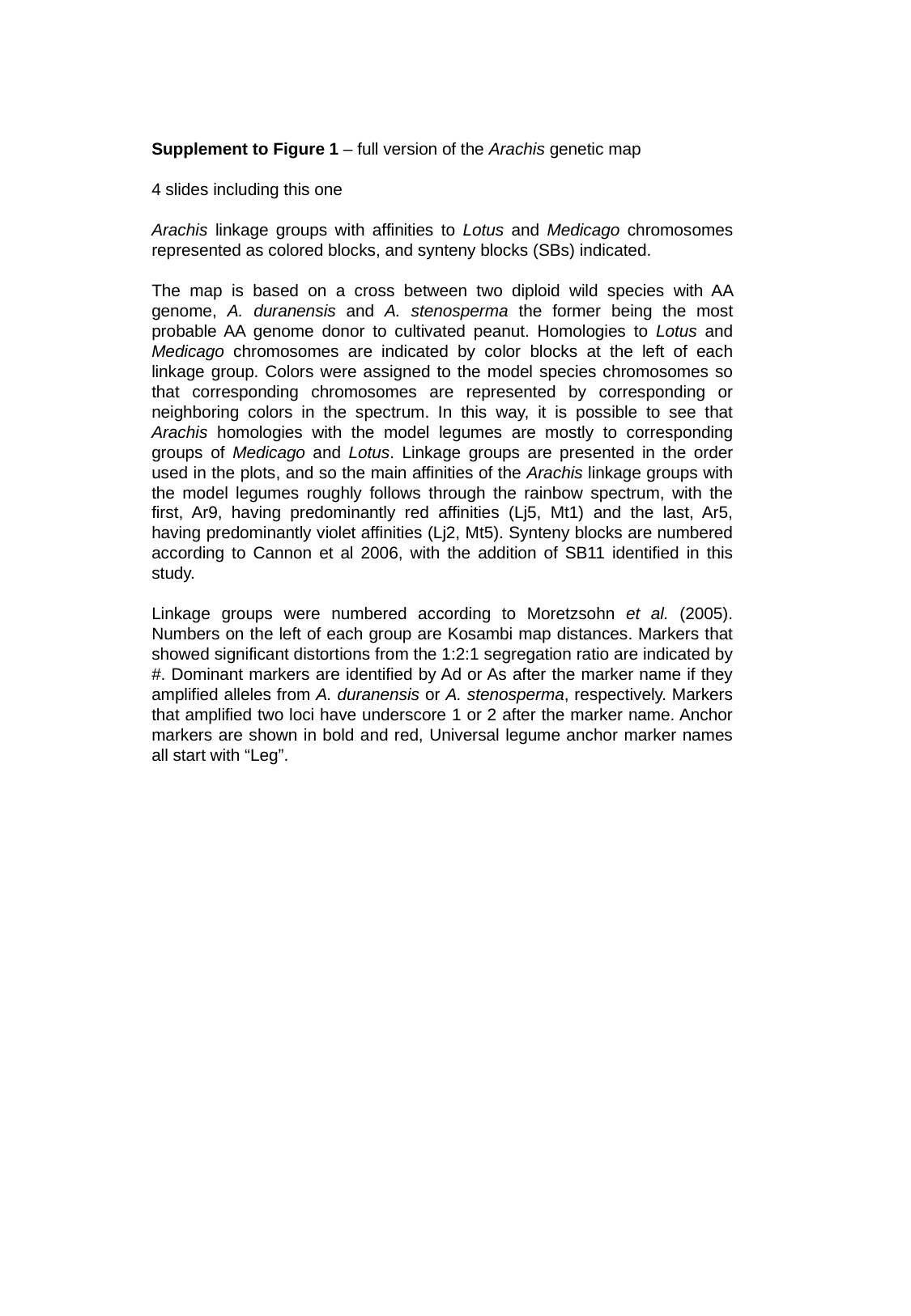

Supplement to Figure 1 – full version of the Arachis genetic map
4 slides including this one
Arachis linkage groups with affinities to Lotus and Medicago chromosomes represented as colored blocks, and synteny blocks (SBs) indicated.
The map is based on a cross between two diploid wild species with AA genome, A. duranensis and A. stenosperma the former being the most probable AA genome donor to cultivated peanut. Homologies to Lotus and Medicago chromosomes are indicated by color blocks at the left of each linkage group. Colors were assigned to the model species chromosomes so that corresponding chromosomes are represented by corresponding or neighboring colors in the spectrum. In this way, it is possible to see that Arachis homologies with the model legumes are mostly to corresponding groups of Medicago and Lotus. Linkage groups are presented in the order used in the plots, and so the main affinities of the Arachis linkage groups with the model legumes roughly follows through the rainbow spectrum, with the first, Ar9, having predominantly red affinities (Lj5, Mt1) and the last, Ar5, having predominantly violet affinities (Lj2, Mt5). Synteny blocks are numbered according to Cannon et al 2006, with the addition of SB11 identified in this study.
Linkage groups were numbered according to Moretzsohn et al. (2005). Numbers on the left of each group are Kosambi map distances. Markers that showed significant distortions from the 1:2:1 segregation ratio are indicated by #. Dominant markers are identified by Ad or As after the marker name if they amplified alleles from A. duranensis or A. stenosperma, respectively. Markers that amplified two loci have underscore 1 or 2 after the marker name. Anchor markers are shown in bold and red, Universal legume anchor marker names all start with “Leg”.

## Slide 2
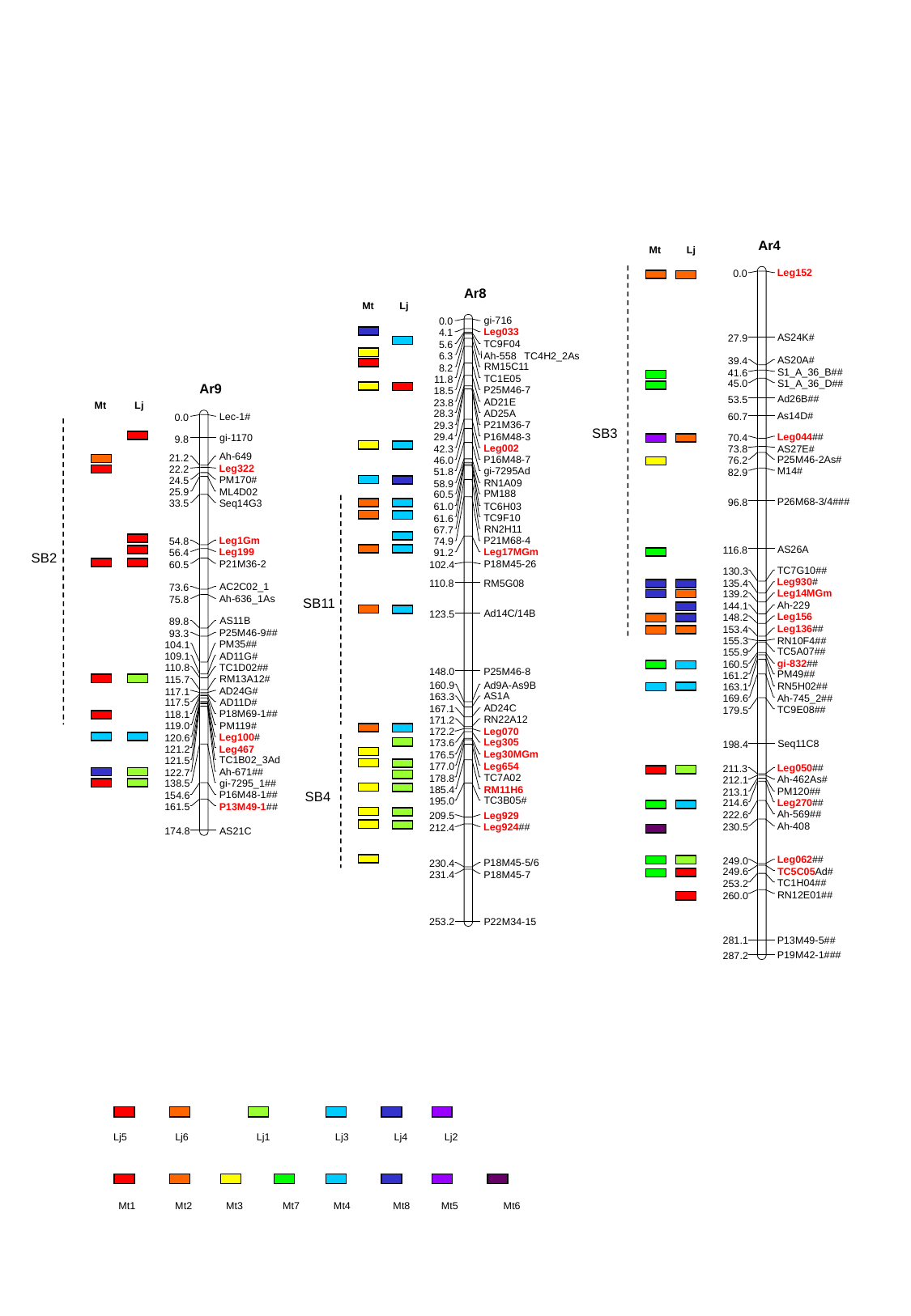

Ar4
Leg152
0.0
AS24K#
27.9
AS20A#
39.4
S1_A_36_B##
41.6
S1_A_36_D##
45.0
Ad26B##
53.5
As14D#
60.7
Leg044##
70.4
73.8
AS27E#
P25M46-2As#
76.2
M14#
82.9
P26M68-3/4###
96.8
AS26A
116.8
TC7G10##
130.3
Leg930#
135.4
Leg14MGm
139.2
Ah-229
144.1
Leg156
148.2
Leg136##
153.4
155.3
RN10F4##
TC5A07##
155.9
gi-832##
160.5
PM49##
161.2
RN5H02##
163.1
169.6
Ah-745_2##
TC9E08##
179.5
Seq11C8
198.4
Leg050##
211.3
Ah-462As#
212.1
PM120##
213.1
214.6
Leg270##
Ah-569##
222.6
Ah-408
230.5
Leg062##
249.0
249.6
TC5C05Ad#
TC1H04##
253.2
RN12E01##
260.0
281.1
P13M49-5##
P19M42-1###
287.2
Mt Lj
SB3
Ar8
Mt Lj
gi-716
0.0
Leg033
4.1
TC9F04
5.6
6.3
Ah-558
 TC4H2_2As
RM15C11
8.2
TC1E05
11.8
P25M46-7
18.5
AD21E
23.8
AD25A
28.3
P21M36-7
29.3
29.4
P16M48-3
Leg002
42.3
P16M48-7
46.0
gi-7295Ad
51.8
RN1A09
58.9
PM188
60.5
TC6H03
61.0
TC9F10
61.6
RN2H11
67.7
P21M68-4
74.9
Leg17MGm
91.2
P18M45-26
102.4
110.8
RM5G08
SB11
Ad14C/14B
123.5
148.0
P25M46-8
160.9
Ad9A-As9B
AS1A
163.3
AD24C
167.1
RN22A12
171.2
Leg070
172.2
Leg305
173.6
Leg30MGm
176.5
177.0
Leg654
TC7A02
178.8
SB4
RM11H6
185.4
TC3B05#
195.0
Leg929
209.5
Leg924##
212.4
P18M45-5/6
230.4
231.4
P18M45-7
P22M34-15
253.2
Ar9
Lec-1#
0.0
gi-1170
9.8
Ah-649
21.2
Leg322
22.2
PM170#
24.5
ML4D02
25.9
33.5
Seq14G3
Leg1Gm
54.8
Leg199
56.4
P21M36-2
60.5
AC2C02_1
73.6
Ah-636_1As
75.8
AS11B
89.8
P25M46-9##
93.3
PM35##
104.1
AD11G#
109.1
110.8
TC1D02##
RM13A12#
115.7
AD24G#
117.1
AD11D#
117.5
P18M69-1##
118.1
119.0
PM119#
Leg100#
120.6
Leg467
121.2
TC1B02_3Ad
121.5
Ah-671##
122.7
gi-7295_1##
138.5
P16M48-1##
154.6
161.5
P13M49-1##
AS21C
174.8
Mt Lj
SB2
Lj5 Lj6 Lj1 Lj3 Lj4 Lj2
Mt1 Mt2 Mt3 Mt7 Mt4 Mt8 Mt5 Mt6

## Slide 3
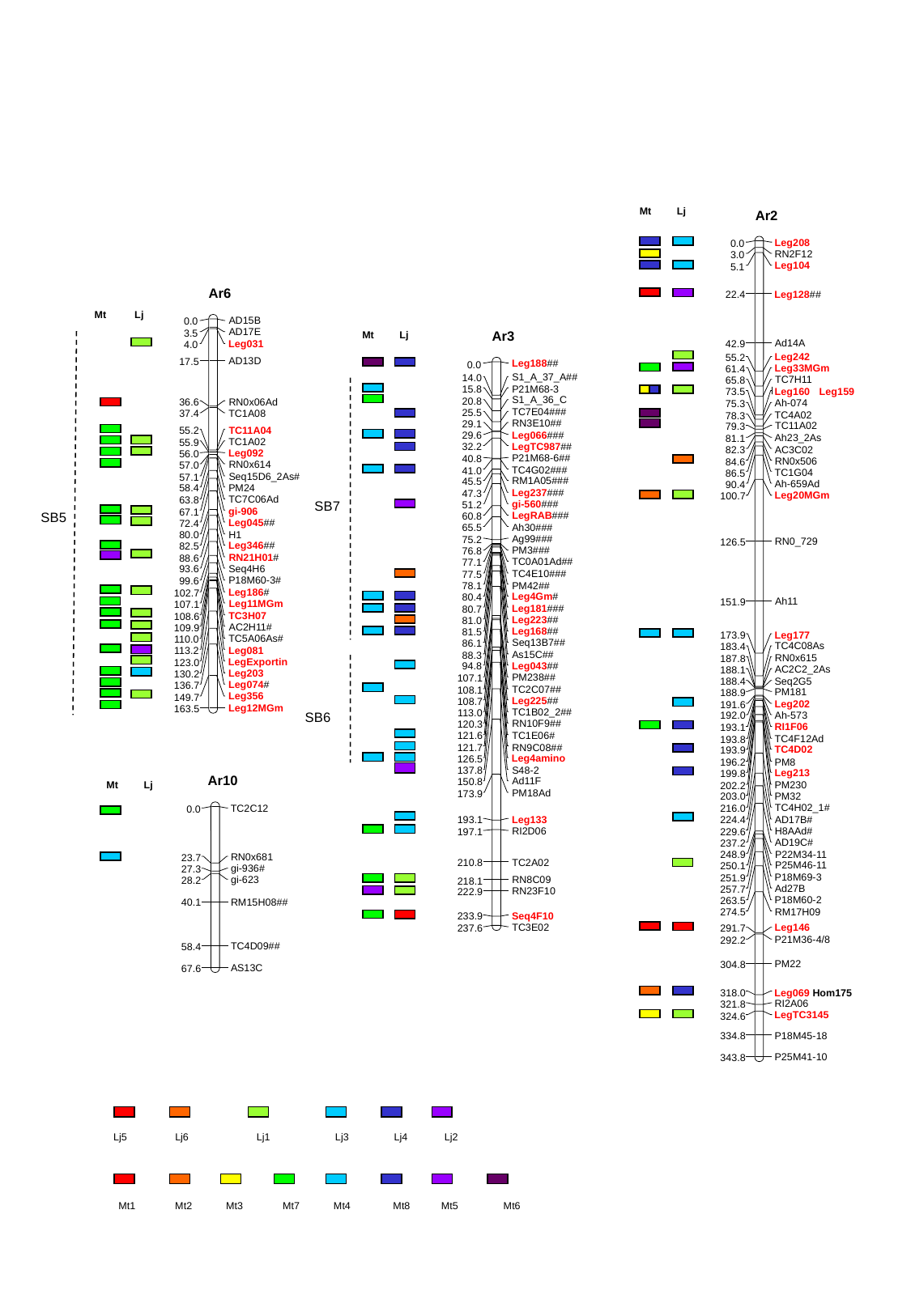

Mt Lj
Ar2
Leg208
0.0
RN2F12
3.0
Leg104
5.1
22.4
Leg128##
Ad14A
42.9
Leg242
55.2
Leg33MGm
61.4
TC7H11
65.8
73.5
Leg160
 Leg159
Ah-074
75.3
TC4A02
78.3
TC11A02
79.3
Ah23_2As
81.1
82.3
AC3C02
RN0x506
84.6
TC1G04
86.5
Ah-659Ad
90.4
Leg20MGm
100.7
RN0_729
126.5
Ah11
151.9
173.9
Leg177
TC4C08As
183.4
RN0x615
187.8
AC2C2_2As
188.1
Seq2G5
188.4
PM181
188.9
Leg202
191.6
192.0
Ah-573
RI1F06
193.1
TC4F12Ad
193.8
TC4D02
193.9
PM8
196.2
Leg213
199.8
PM230
202.2
203.0
PM32
TC4H02_1#
216.0
AD17B#
224.4
H8AAd#
229.6
AD19C#
237.2
248.9
P22M34-11
P25M46-11
250.1
P18M69-3
251.9
Ad27B
257.7
P18M60-2
263.5
RM17H09
274.5
Leg146
291.7
P21M36-4/8
292.2
PM22
304.8
318.0
Leg069 Hom175
RI2A06
321.8
LegTC3145
324.6
P18M45-18
334.8
P25M41-10
343.8
Ar6
AD15B
0.0
AD17E
3.5
Leg031
4.0
AD13D
17.5
36.6
RN0x06Ad
TC1A08
37.4
55.2
TC11A04
TC1A02
55.9
Leg092
56.0
RN0x614
57.0
Seq15D6_2As#
57.1
58.4
PM24
TC7C06Ad
63.8
gi-906
67.1
Leg045##
72.4
H1
80.0
Leg346##
82.5
RN21H01#
88.6
93.6
Seq4H6
P18M60-3#
99.6
Leg186#
102.7
Leg11MGm
107.1
TC3H07
108.6
AC2H11#
109.9
TC5A06As#
110.0
113.2
Leg081
LegExportin
123.0
Leg203
130.2
Leg074#
136.7
Leg356
149.7
Leg12MGm
163.5
Mt Lj
SB5
Mt Lj
Ar3
Leg188##
0.0
S1_A_37_A##
14.0
P21M68-3
15.8
S1_A_36_C
20.8
TC7E04###
25.5
RN3E10##
29.1
Leg066###
29.6
32.2
LegTC987##
P21M68-6##
40.8
TC4G02###
41.0
RM1A05###
45.5
Leg237###
47.3
gi-560###
51.2
LegRAB###
60.8
65.5
Ah30###
Ag99###
75.2
PM3###
76.8
TC0A01Ad##
77.1
TC4E10###
77.5
78.1
PM42##
Leg4Gm#
80.4
Leg181###
80.7
Leg223##
81.0
Leg168##
81.5
Seq13B7##
86.1
As15C##
88.3
94.8
Leg043##
PM238##
107.1
TC2C07##
108.1
Leg225##
108.7
TC1B02_2##
113.0
RN10F9##
120.3
TC1E06#
121.6
121.7
RN9C08##
Leg4amino
126.5
S48-2
137.8
Ad11F
150.8
PM18Ad
173.9
193.1
Leg133
RI2D06
197.1
210.8
TC2A02
RN8C09
218.1
RN23F10
222.9
233.9
Seq4F10
TC3E02
237.6
SB7
SB6
Mt Lj
Ar10
TC2C12
0.0
RN0x681
23.7
gi-936#
27.3
gi-623
28.2
RM15H08##
40.1
TC4D09##
58.4
AS13C
67.6
Lj5 Lj6 Lj1 Lj3 Lj4 Lj2
Mt1 Mt2 Mt3 Mt7 Mt4 Mt8 Mt5 Mt6

## Slide 4
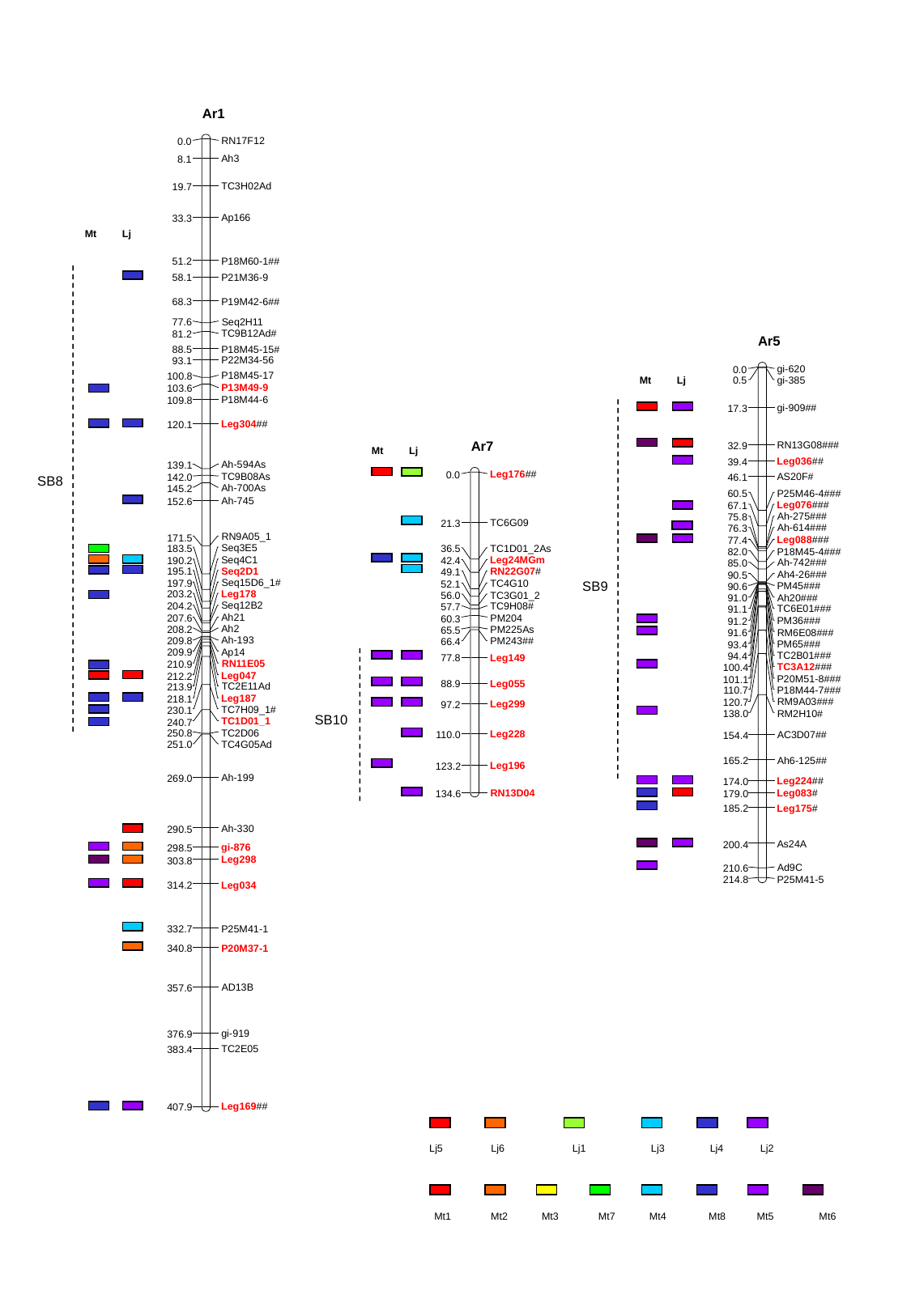

Ar1
RN17F12
0.0
Ah3
8.1
TC3H02Ad
19.7
Ap166
33.3
P18M60-1##
51.2
P21M36-9
58.1
68.3
P19M42-6##
Seq2H11
77.6
TC9B12Ad#
81.2
P18M45-15#
88.5
P22M34-56
93.1
P18M45-17
100.8
P13M49-9
103.6
P18M44-6
109.8
Leg304##
120.1
Ah-594As
139.1
TC9B08As
142.0
Ah-700As
145.2
Ah-745
152.6
RN9A05_1
171.5
Seq3E5
183.5
Seq4C1
190.2
Seq2D1
195.1
Seq15D6_1#
197.9
203.2
Leg178
Seq12B2
204.2
Ah21
207.6
Ah2
208.2
Ah-193
209.8
209.9
Ap14
RN11E05
210.9
Leg047
212.2
TC2E11Ad
213.9
Leg187
218.1
TC7H09_1#
230.1
TC1D01_1
240.7
250.8
TC2D06
TC4G05Ad
251.0
Ah-199
269.0
Ah-330
290.5
gi-876
298.5
Leg298
303.8
314.2
Leg034
332.7
P25M41-1
340.8
P20M37-1
AD13B
357.6
gi-919
376.9
TC2E05
383.4
Leg169##
407.9
Mt Lj
Ar5
gi-620
0.0
gi-385
0.5
gi-909##
17.3
RN13G08###
32.9
Leg036##
39.4
AS20F#
46.1
60.5
P25M46-4###
Leg076###
67.1
Ah-275###
75.8
Ah-614###
76.3
Leg088###
77.4
82.0
P18M45-4###
Ah-742###
85.0
Ah4-26###
90.5
PM45###
90.6
Ah20###
91.0
TC6E01###
91.1
PM36###
91.2
91.6
RM6E08###
PM65###
93.4
TC2B01###
94.4
TC3A12###
100.4
P20M51-8###
101.1
P18M44-7###
110.7
RM9A03###
120.7
138.0
RM2H10#
AC3D07##
154.4
165.2
Ah6-125##
Leg224##
174.0
Leg083#
179.0
185.2
Leg175#
As24A
200.4
Ad9C
210.6
214.8
P25M41-5
Mt Lj
SB9
Ar7
Leg176##
0.0
TC6G09
21.3
TC1D01_2As
36.5
Leg24MGm
42.4
RN22G07#
49.1
TC4G10
52.1
56.0
TC3G01_2
TC9H08#
57.7
PM204
60.3
PM225As
65.5
PM243##
66.4
Leg149
77.8
Leg055
88.9
Leg299
97.2
Leg228
110.0
Leg196
123.2
RN13D04
134.6
Mt Lj
SB8
SB10
Lj5 Lj6 Lj1 Lj3 Lj4 Lj2
Mt1 Mt2 Mt3 Mt7 Mt4 Mt8 Mt5 Mt6
